# Augmented antibiotic resistance associated with cadmium induced alterations in *Salmonella enterica* serovar Typhi

**DOI:** 10.1038/s41598-018-31143-9

**Published:** 2018-08-24

**Authors:** Ujjwal Jit Kaur, Simran Preet, Praveen Rishi

**Affiliations:** 1 Department of Microbiology, Panjab University, Chandigarh, India; 2 Department of Biophysics, Panjab University, Chandigarh, India

**Keywords:** Reverse transcription polymerase chain reaction, Bacterial pathogenesis, Biofilms, Pathogens

## Abstract

In view of the reports on co-selection of metal and antibiotic resistance, recently we have reported that increased cadmium accumulation in *Salmonella* Typhi Ty2 leads to increased antibiotic resistance. In continuation, the present study was carried to substantiate this association in clinical isolates. Interestingly, the levels of cadmium were found to be more in the clinical isolates which co-related with their antibiotic sensitivity/resistance pattern. On cadmium accumulation, antibiotic(s) sensitive isolates were rendered resistant and the resistant isolates were rendered more resistant as per their minimum inhibitory concentration(s). Further, after subjecting the pathogen to cadmium accumulation, alterations occurring in the cells were assessed. Transgenerational cadmium exposure led to changes in growth response, morphology, proteome, elevated antioxidants other than SOD, increased biofilm formation, decreased intracellular macrophage killing coupled with upregulation of genes encoding metallothionein and metal transporters. Thus, these results indicate that cadmium, if acquired from the environment, being non-degradable can exert a long-lasting selective pressure on *Salmonella* in the host which may display antibiotic resistance later on, as a result of co-selection. Therefore, appropriate strategies need to be developed to inhibit such an enduring pressure of heavy metals, as these represent one of the factors for the emerging antibiotic resistance in pathogens.

## Introduction

Heavy metals are widespread in sewage as a consequence of ungoverned industrial and anthropological activities^1–3^. Thus, in natural habitats, bacteria are continuously exposed to different metals, thereby giving rise to survival of the metal tolerant cells due to mutations^4^. *Salmonella* is known to persist for longer period in sludge of sewage treatment plant (STP) where it encounters the heavy metal selective pressure^5^. The pathogen penetrates into the food chain and water through agricultural practises where effluents and minimally treated sewage sludge are used in order to recirculate nutrients from sludge to arable land, thereafter entering the human host through contaminated food and water^6,7^.

It has been well documented that active transporters participating in the regulation of influx and efflux systems of the organisms account for adaptations to metals present in the environment^8,9^. Genes determining metal tolerance as well as antibiotic resistance may be located either on the same genetic structure (eg. plasmid) or different genetic structure within same bacterial strain^1^ thus affecting the regulation and expression of each other. Significantly enhanced expression of genes involved in co-selection/co-inheritance of metal and antibiotic resistance has been reported in various pathogens including *Escherichia*. *coli*, *Pseudomonas*, *Acinetobacter* and *Listeria*^10–15^. The genes that are responsible for tolerance to arsenic, copper and zinc have been observed to be present in methicillin resistant *Staphylococcus aureus* isolated from livestock^16^. The same has also been suggested for *Salmonella* Typhimurium isolated from retail foods^17^. Unlike antibiotics, metals are not subjected to degradation and can subsequently represent an enduring selection pressure^18,19^. Thus, there are concerns regarding the potential of metal contamination in maintaining a pool of antibiotic resistance genes in both environmental as well as clinical settings.

*Salmonella enterica* serovar Typhi (the causative agent of typhoid fever) which enters the host via feco-oral route represents a major health concern and is associated with the number of epidemics. *Salmonella* already exposed to heavy metals in the natural environment may cause the pathogen to become resistant to the advocated antibiotics inside the host as per the co-selection theory. Recently we have reported that intracellular accumulation of cadmium in *Salmonella enterica* serovar Typhi Ty2 (reference strain) leads to increased antibiotic resistance^20^. In continuation to this report, the present study was carried out to validate the cadmium-antibiotic co-relationship in the antibiotic resistant clinical isolates and to evaluate cadmium induced alterations in the pathogen contributing to this association.

## Materials and Methods

### Bacterial strains

*S*. *enterica* serovar Typhi Ty2, reference strain (DBL-8, David Bruce Laboratory, East Everleigh, Marlborough Wiltshire) was originally procured from Central Research Institute, Kasauli. It has been maintained in our laboratory^21^. The clinical isolates of serovar Typhi procured earlier from All India Institute of Medical Sciences (AIIMS), New Delhi; Maulana Azad Medical College (MAMC), New Delhi and Government Medical College and Hospital, Chandigarh for epidemiological studies, were used in the present study. These isolates were recovered from random, sporadic and unrelated cases of typhoid fever which belonged to different clusters^22^. All the isolates were preserved in 40% glycerol stocks and stored at −80 °C.

### Agents

Ampicillin, ciprofloxacin, chloramphenicol, ceftizoxime and TRIZOL reagent were procured from Sigma-Aldrich (St Louis, MO, USA). Cadmium chloride (CdCl_2_) salt was procured from Sisco Research Laboratories Pvt. Ltd. (Mumbai). cDNA reaction kit and 2X iQ SYBR Green Supermix were purchased from Bio-Rad Laboratories (India) Pvt. Ltd. The antibiotics were stored as standard stocks of 1 g/L at −20 °C.

### Flame atomic absorption spectroscopy (FAAS)

The intracellular concentration(s) of various metals like Zn, Ni, Cu, Co, Pb, Mn, Fe, Ca, Cd in all the serovar Typhi isolates was assessed using AA- 6800, Shimadzu flame atomic absorption spectrophotometer (FAAS). The metals were extracted out using HNO_3_ and perchloric acid which oxidises the organic component of the samples^20,23^.

### Determination of minimum inhibitory concentration (MIC)

MICs of ampicillin, ciprofloxacin, chloramphenicol and ceftizoxime against reference as well as clinical isolates before and after adaptation (described below) were determined by broth dilution techniques (CLSI, 2012), as described by us earlier^24^.

### Adaptation of reference strain and clinical isolates of *S*. *enterica* serovar Typhi isolates to cadmium

Cadmium adapted (CdA) serovar Typhi cells were obtained by carrying out sequential propagation (ten passages) in nutrient broth supplemented with cadmium chloride at its sub-MIC (0.5 mM) under laboratory conditions, as described by us earlier^20^. In addition, all the parameters regarding the bioavailability of cadmium chloride in the medium were taken into consideration.

### Characterisation of CdA Salmonellae

The CdA serovar Typhi isolates were quantitatively characterised by (i) FAAS analysis by estimating the intracellular cadmium concentration as mentioned earlier^20^. The fold change in accumulation in CdA cells with respect to cadmium unadapted (CdunA) cells was recorded in terms of ppm (mg/L). ANOVA single factor (one way analysis of variance) was performed keeping altered antibiotic MIC values as dependent variable and cadmium accumulation, antibiotic types as independent variables. (ii) The growth response of CdA and CdunA Ty2 cells in nutrient broth (beef extract-1g/L, peptone- 5 g/L, yeast extract-2 g/L, NaCl-5g/L) was also monitored. Briefly, 1% (10^6^ colony forming units/ml) of overnight grown bacterial culture was inoculated in nutrient broth and incubated at 37 °C under shaking conditions. O.D. 600 nm and log_10_ CFU/ml were recorded at different time intervals for 48 h. (iii) Additionally, the CdA Ty2 cells were characterised by observing cadmium associated ultrastructural alterations using transmission electron microscopic (TEM) analysis.

### Mechanistic studies

#### Cadmium associated phenotypic alterations in reference Ty2 strain

Proteome analysis: Total proteins from the CdA as well as CdunA cells were extracted^25^ and fractionated into cytoplasmic (CYT), inner membrane (IM) and outer membrane fraction (OM) and visualized using SDS- PAGE. GelQuant (DNR Bio-Imaging Systems Ltd.) and ImageJ were used for image analysis to estimate the approximate molecular weight of differentially/over expressed proteins and fold increase in the expression of proteins.

Native gel zymography of SOD: a metal associated enzyme: The in-gel SOD activity was evaluated by native-PAGE zymography i.e. after electrophoresis the gel was soaked in NBT-riboflavin solution as described by us earlier^20^ and the SOD isoforms in both the CdunA and CdA Ty2 cells were compared.

Biochemical analysis: Overall estimation of cellular antioxidants like superoxide dismutase (SOD), catalase (CAT), reduced glutathione (GSH), glutathione reductase (GR), ascorbate peroxidase (APOX) activity and non-protein thiol (NPSH) content was performed in both the CdunA and CdA Ty2 cells by the protocols of Shamim and Rehman^26^.

Evaluation of biofilm forming potential: Biofilms of CdA and CdunA Ty2 cells at cell density of 10^7^ colony forming units/ml were established in 96-well microtiter plate according to the method of Wong *et al*.^27^ and Peng^28^, with some modification. The optical density and log_10_ colony forming units/ml in the biofilms were determined after 24, 48 and 72 h.

Macrophage intracellular survival assay: For extracting the murine macrophages, the guidelines issued by the Institutional Animal Ethics Committe Panjab University, Chandigarh (India) were followed. Peritoneal macrophages from mice were extracted and infected with CdunA and CdA Ty2 cells at a multiplicity of infection of 1:100 (macrophage: bacteria), as described by us earlier^29,30^.

qRT- PCR of *smtA* and *ybaL* genes: Differential gene expression of *smtA* gene (encodes for metallothionein- a metal binding protein) and *ybaL* gene (cation: proton antiport protein) was determined in CdA Ty2 cells using real time PCR studies. *smtA* (Forward primer: 5′-CAAAGGACAACTGCGGCAAG-3′ and Reverse Primer: 5′-ATAACGTCACCTGATGGCCG-3′) and *ybaL* (Forward Primer: 5′-CCGTGCTGGGATGGTCATTA-3′ and Reverse Primer: 5′-CGACATCGCCTTTTTCCACC-3′) were synthesised from gene sequences (*smtA*- Gene ID: 1252509 and *ybaL*- Gene ID: 1247009) available in NCBI database using NCBI primer designing tool. The PCR protocol used on Applied Biosystem step one real time PCR system started with the first step of initial denaturation and enzyme activation at 95 °C for 3 min, followed by 40 cycles of denaturation at 95 °C for 10 s, annealing and extension at 55 °C for 1 min. Melt curve analysis was performed by heating the samples from 55 °C to 95 °C with an increment of 0.5 °C and fluorescence was recorded. Under these experimental conditions, GAPDH was used as reference gene.

### Statistical analysis

Data were expressed as mean ± standard deviation of three independent experiments. Statistical data analysis was done using SPSS 16.2 for Windows (SPSS Inc., Chicago, IL) and GraphPad Prism 5 software by evaluating significance of data using Student’s two sample t-test and one way analysis of variance (ANOVA). During data analysis, *p*-values of 0.05 or less (*p* < 0.05) were considered significant.

## Results and Discussion

An indirect link between metal and antibiotic resistance has been reported in various microorganisms^10–16^. Metal ions can function in the selective propagation of antibiotic resistant microorganisms in environmental as well as clinical settings^3^. A recent observation from our laboratory^20^ that intracellular cadmium plays a role in the antibiotic sensitivity pattern of *Salmonella*, prompted us to study this association in the clinical isolates along with its effect on other metal associated changes in *S*. Typhi.

In the present study, when different metals were estimated in clinical isolates, cadmium was found to be predominantly present as observed previously in the reference strain. On analysing the antibiotic susceptibility pattern of the isolates, it was observed that the resistant isolates had more cadmium levels as compared to the levels present in the sensitive isolates (Table 1). This surprisingly high concentration of cadmium can be attributed to relative higher exposure of *Salmonella* in metal contaminated sewage water, as metals are not subjected to degradation which subsequently represents an enduring selection pressure^31^. Incorporation of this non-essential active metal in sub-cellular compartments might be due to its “look alike” nature to calcium and zinc (required by the bacterial cells for various metabolic processes)^32^.

**Table 1:** Change in MICs and cadmium accumulation of the reference as well as the clinical isolates of *Salmonella enterica* serovar Typhi upon cadmium exposure.

Isolate	Cadmium Conc. (ppm or mg/L)		Fold increase in Metal Conc.	MIC (mg/L)								Fold increase in MIC
				Ampicillin		Ciprofloxacin		Chloramphenicol		Ceftizoxime		
	NM^a^	M^b^		NM	M^@^	NM	M^@^	NM	M^@^	NM	M^@^	
Ty2	0.691	9.610	13.9	4	16^**^	0.5	1^**^	2	8^**^	0.0625	0.0625	4, 2, 4, 1
18G	1.482	9.405	6.346	2	2	16	32^**^	8	8	0.0625	0.125^**^	1, 2, 1, 2
35G	1.224	8.039	6.6	2	4^**^	8	18^**^	4	4	0.03125	0.03125	2, 2.2, 1.5, 1
17G	1.219	6.435	5.3	2	8^**^	16	32^**^	4	8^**^	0.0625	0.0625	4, 2, 2, 1
6G	1.280	4.102	3.204	2	2	4	8^**^	4	4	0.03125	0.0625^**^	1, 2, 1, 2
20A	1.188	6.093	5.128	2	2	0.5	1^**^	4	4	0.125	0.125	1, 2, 1, 2
20C	0.583	3.102	5.320	0.5	0.5	0.5	2^**^	1	2^**^	0.03125	0.0625^**^	1, 4, 2, 2
12G	0.817	13.246	16.2129	0.5	4^**^	8	20^**^	2	4^**^	0.0625	0.25^**^	8, 2.5, 2, 4
21G	1.247	10.187	8.169	2	4^**^	2	16^**^	4	4	0.03125	0.125^**^	2, 8, 1, 4
16C	1.437	10.296	7.1649	8	12	2	10^**^	8	10	0.0625	0.0625	1.5, 5, 1.25, 1
25C	0.594	11.781	19.833	0.5	2^**^	1	1	2	4^**^	0.0625	0.125^**^	4, 1, 2, 2
9G	1.325	9.719	7.33	2	4^**^	16	30	16	25	0.03125	0.0625^**^	2, 1.9, 1.625, 2
11C	1.350	7.424	5.499	16	20	0.5	2^**^	8	8	0.0625	0.25^**^	1.25, 4, 1, 4
12C	1.412	6.710	4.7521	8	16^**^	0.5	2^**^	8	10	0.0625	0.125^**^	2, 4, 1.25, 2
14C	1.392	8.779	6.307	8	8	1	4^**^	4	4	0.0625	0.125^**^	1, 4, 1.25, 2
15C	1.420	16.489	11.612	8	8	1	8^**^	8	10	0.0625	0.25^**^	1, 8, 1.25, 4
7G	1.297	19.844	10.67	2	4^**^	4	16^**^	4	4	0.03125	0.25^**^	2, 4, 1, 8
19A	1.152	5.915	5.135	256	256	0.5	8^**^	32	32	0.25	0.25	1, 16, 1, 1
17A	1.110	12.496	11.257	256	256	0.5	0.5	32	32	0.03125	0.03125	1, 1, 1, 1
18A	1.149	9.148	7.9617	1	1	0.5	0.5	4	4	0.06125	0.06125	1, 1, 1, 1
21C	1.515	23.968	15.82	8	8	1	8^**^	4	6	0.0625	0.0625	1, 8, 1.5, 1
16G	1.298	9.753	7.51	4	4	32	36	2	2	0.125	0.125	1, 1.125, 1, 1
46G	1.482	5.055	3.4166	2	8^**^	2	16^**^	4	4	0.0625	0.0625	4, 8, 1, 1
3m	1.515	14.237	9.393	2	4^**^	2	32^**^	4	4	0.125	0.25^**^	2, 16, 1, 2
4m	1.529	28.13	18.4	2	2	2	2	8	12	0.03125	0.0625^**^	1, 1, 1.375, 2
19C	0.65	10.8	16.615	4	16^**^	0.5	1^**^	2	8^**^	0.0625	0.0625	4, 2, 4, 1
18C	0.62	10.1	16.290	0.5	2^**^	1	1	2	4^**^	0.0625	0.125^**^	4, 1, 2, 2
10G	0.58	11.3	19.48	4	16^**^	1	1	2	8^**^	0.0625	0.125^**^	4, 1, 4, 2
11G	0.61	11.1	18	0.5	2^**^	0.5	1^**^	2	4^**^	0.0625	0.0625	4, 2, 2, 1

(^a^NM- Non-metal exposed cells, ^b^M- Metal adapted cells, ^**^2 0r above 2 fold change in MIC of antibiotics). Cut-off values of different antibiotics according to CLSI guidelines: Ampicillin- ≤ 8, 16, ≥32; Ciprofloxacin- ≤ 0.06, 0.125–0.5, ≥1; Chloramphenicol- ≤ 8, 16, ≥32; Ceftizoxime- ≤ 1, 2, ≥4. ^@^*p* ≤ 0.05 versus heavy metal accumulation.

These observations made us speculate that higher concentration of cadmium in the clinical isolates (Table 1) may be one of the factors for the antibiotic resistance. Therefore, to further acertain the role of cadmium, clinical *Salmonella* isolates were adapted to sub-MIC (0.5 mM) of cadmium chloride for ten subsequent generations, under laboratory conditions in order to attain the maximum permissible level that can be tolerated by the organism. Re- FAAS analysis confirmed the increase in the intracellular cadmium content in all the isolates (Table 1). These results are in agreement with previous results of the reference CdA Ty2 strain, where 13.9 fold increase in cadmium content was observed^20^. In the present study, cadmium adaptation in serovar Typhi isolates (19C, 18C, 12G, 11G, 10G and 25C) exhibiting resistance to least one antibiotic revealed as high as 16.61, 16.29, 16.22, 18.0, 19.48 and 19.83 fold increase in intracellular cadmium content. This observation highlighted the fact that the isolates which initially had low cadmium levels demonstrated higher intracellular cadmium accumulation upon exposure to cadmium chloride (Table 1). The analysis of variance (ANOVA) indicated the association in the change in MIC of antibiotics and cadmium accumulation (*p* < 0.05).

Antibiogram analysis of CdA serovar Typhi isolates revealed that the sensitive isolates became resistant to antibiotics as per their MICs, as compared to MICs observed before adaptation (Table 1) and these observations are in concordance with our previous observations^20^. Overall in clinical isolates, cadmium induced MIC variation of ciprofloxacin was more frequent (76% isolates) followed by ceftizoxime (55% isolates), ampicillin (55% isolates) and chloramphenicol (36% isolates) (Fig. S2). Thus, with overall augmentation in the antibiotic MICs, sensitive serovar Typhi isolates became resistant and resistant isolates became more resistant to antibiotics upon cadmium accumulation (Table 1) indicating a co-association of antibiotic resistance and presence of the metal. Bacterial resistance to metals and antibiotics are often genetically linked and exposure to metal, may select for strains resistant to antibiotics and vice-versa^31^. Increased antibiotic resistance might be attributed to alteration in the efflux or the metal sequestration mechanisms as well as biofilm forming potential as a result of involvement of a co-regulatory response to metal and antibiotic stress. A range of transcriptional and translational responses to metal or antibiotic exposure can be linked to form a coordinated response to both the stresses^3^.

**Figure 1:**
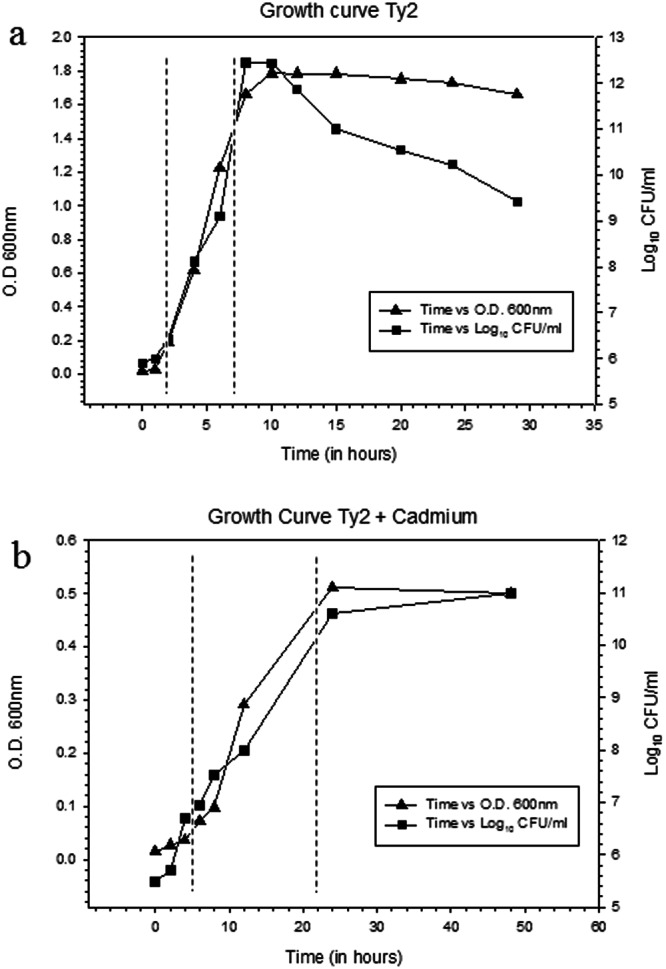
Growth pattern curve showing optical density at 600 nm and log_10_ CFU/ml along the vertical axis at different time intervals.

**Figure 2:**
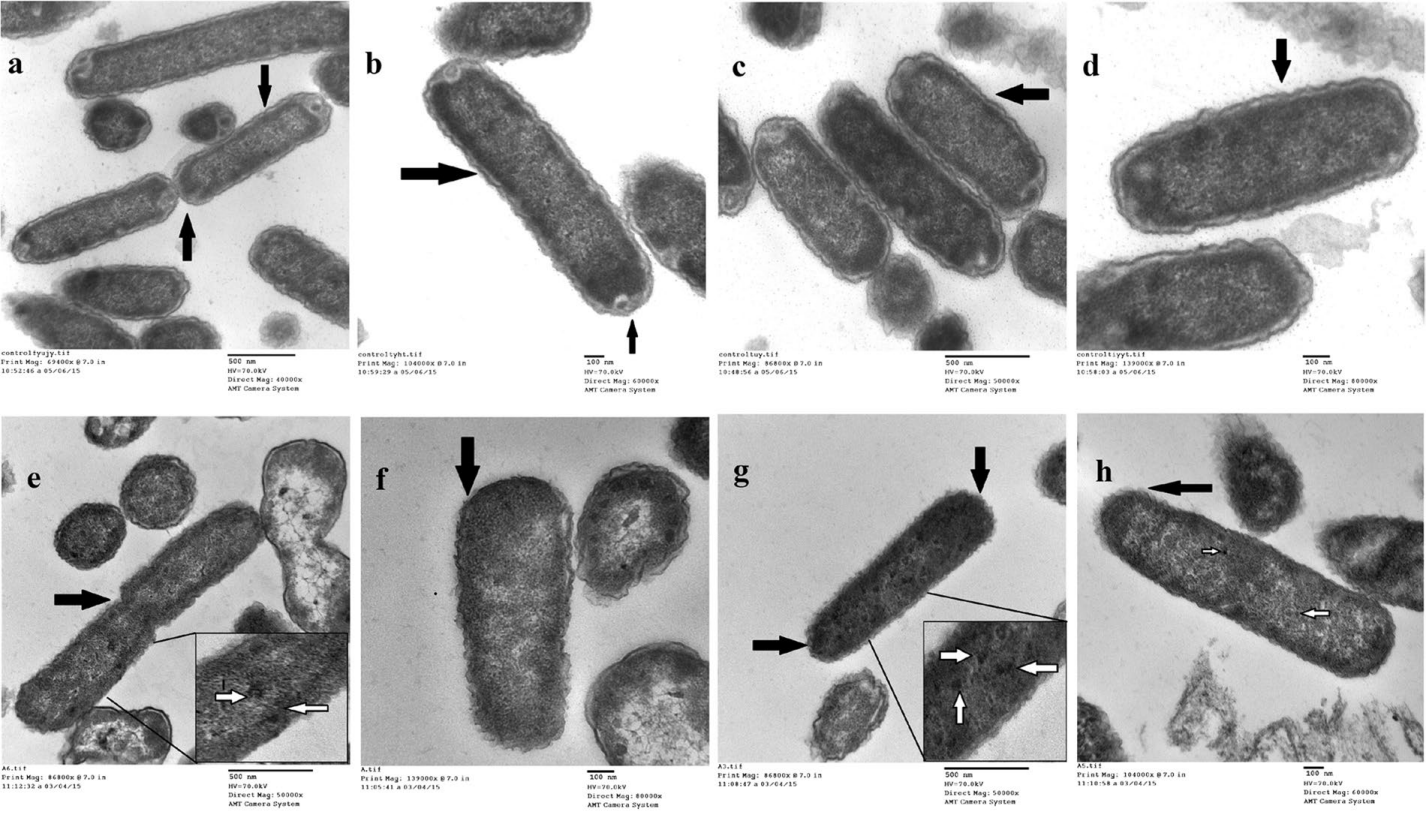
Transmission electron micrographs of reference serovar Typhi Ty2 strain. **(a**–**d)** Normal *Salmonella* cells and **(e**–**h)** cadmium adapted *Salmonella* cells. Arrows highlights the differences observed. Inset- White arrows marked the electron dense regions in the enhanced micrographic image.

Further, in order to elucidate the changes occurring in the cells on cadmium adaptation, CdA Ty2 cells were characterized. At 0.5 mM cadmium chloride supplementation, no visible precipitation or any other change, except small shift in pH i.e. from 7.2 to 7.0 was observed suggesting that the bioavailability of cadmium in nutrient broth was not limited, which is concordance with previous reports where nutrient broth supplemented with cadmium chloride had been used in order to study metal induced stress in bacteria^33–36^. Re-FAAS analysis revealed the increased levels of cadmium as observed in the clinical isolates. Growth response of CdA Ty2 cells was found to be strikingly different from CdunA Ty2 cells (Fig. 1). A marked reduction in the cell density was also observed in CdA Ty2 cells during growth kinetics. A delay in the lag phase and prolonged log phase observed in CdA Ty2 cells may be attributed to the fact that metabolism dependent uptake of metal ions is usually a slower process thereby delaying the lag and log phases^37–40^. To adapt to stresses, bacteria can activate a number of envelope stress responses that sense specific signals and regulate gene expression^41^. The effect of metal stress in CdA Salmonellae was visualised using TEM (Fig. 2) which revealed considerable structural changes, disclosing extensive membrane alterations (intense wavy appearance) and significantly reduced periplasmic space in contrast to CdunA Ty2 cells. Further, stained micrographs of cadmium adapted cells reflected the loss of intracellular organisation with disappearance of certain cellular compartments present towards the polar regions of the stained cells (Fig. 2f–h). Additionally, in the micrographs some prominent electron dense regions (highlighted using inset and white arrows- Fig. 2e–h) ascertained the sequestration of cadmium within CdA serovar Typhi Ty2 cells as suggested earlier^42,43^.

**Figure 3:**
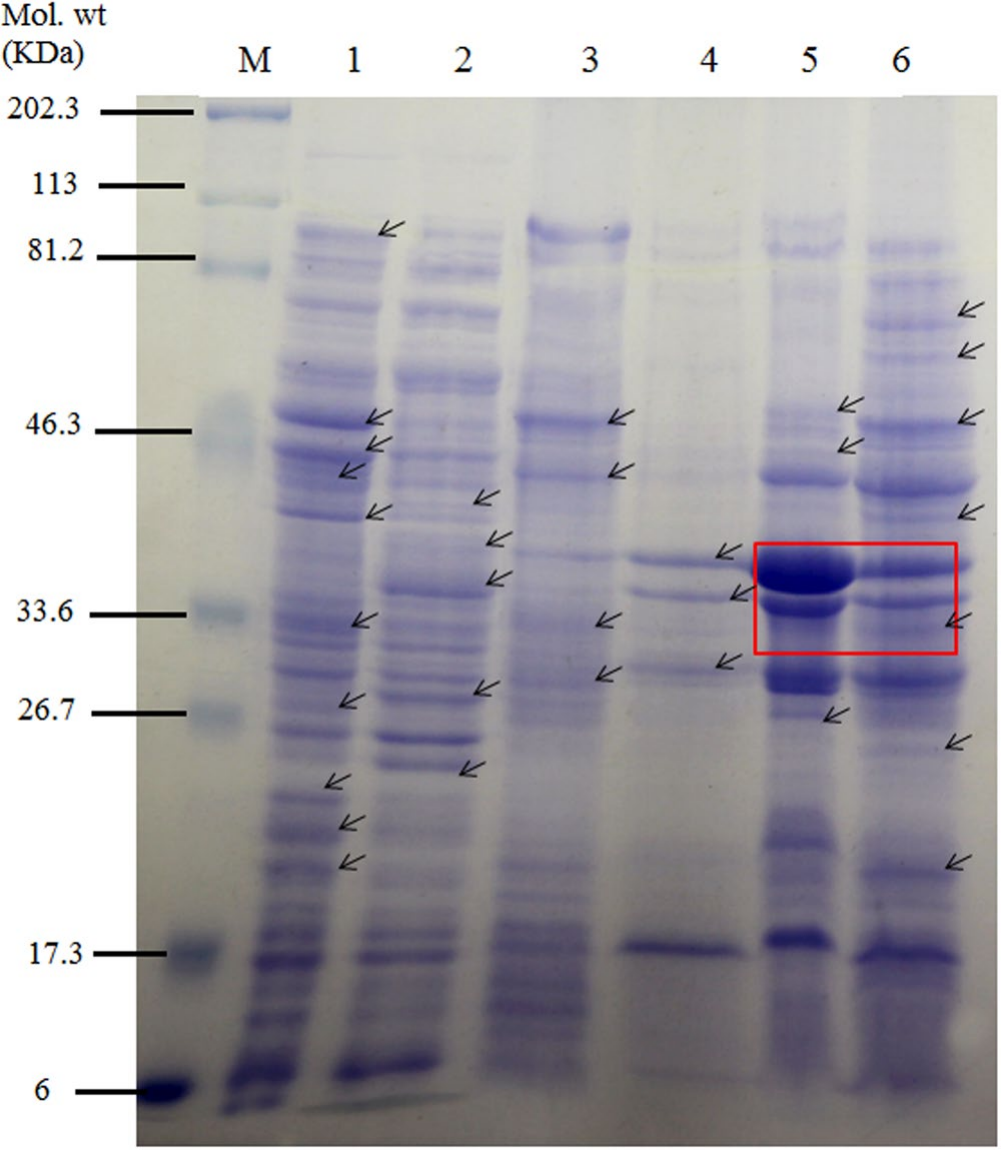
SDS-PAGE analysis of different protein fractions from CdunA serovar Typhi Ty2 cells and CdA Ty2 cells. Lane M- Broad range protein molecular weight marker; Lane 1,3,5- CYT, IM, OM fractions from CdunA Ty2 cells and Lane 2,4,6- CYT, IM, OM fractions from CdA Ty2 cells. Arrows indicate the changes observed. (Full-length gel is presented in Supplementary Fig. S3).

Proteome analysis revealed differential expression of proteins in CdA Salmonellae. A 2.12 fold (relative density) reduction in the expression of porins as per densitometric analysis using ImageJ software was observed which is in agreement with the earlier reports^44–47^. The CdA cells might have down regulated porins thereby inhibiting the influx of the desired molecules or antibiotics (Fig. 3). Comparative analysis of different cellular fractions of the CdA Ty2 strain with CdunA Ty2 strain, revealed a total of 16 significantly expressed proteins (Fig. 3) with molecular weight of (**CYT**-41.6 kDa, 39.0 kDa, 37.8 kDa, 27.6 kDa, 24.3 kDa; **IM**-36.8 kDa, 34.0 kDa, 29.5 kDa; **OM**-67.3 kDa, 60.4 kDa, 46.0 kDa, 40.0 kDa, 32.1 kDa, 25.1 kDa, 20.1 kDa). Representative SDS-PAGE gels for two clinical isolates have also been included in the supplementary file (Figs S4 and S5). We speculate that downregulation of porins, in particular, may contribute as a mode of co-selection of metal and antibiotic resistance in bacteria, by conferring outer membrane permeability barrier^45–47^.

**Figure 4:**
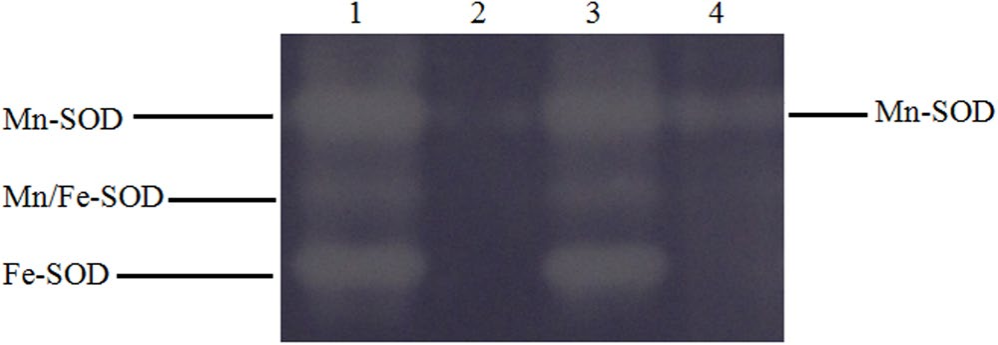
Cadmium dependent variation of SOD enzymes. Serovar Typhi 25 C and 12 G grown with and without cadmium supplementation, whole cell lysates (WCL) were loaded on native PAGE gel and stained to visualise different isoforms of SOD. Lane 1,3 - normal serovar Typhi cells and Lane 2,4 - cadmium adapted serovar Typhi cells. (Full-length gel is presented in Supplementary Fig. S6).

To identify a possible link between cadmium stress and superoxide dismutase activity, we have analysed Mn-SOD, Mn/Fe-SOD and FE-SOD activity in CdA isolates. The native PAGE gel revealed only Mn-SOD to be present in CdA cells as compared to three isoforms observed in CdunA cells. This is in concordance with our previous report on reference strain^20^. Representative gel for two clinical isolates (25C and 12G) has been shown in Fig. 4. It may be inferred that cadmium might have initiated a complete cascade of reactions by releasing free Fe+ ions from SOD which further catalyses the generation of reactive oxygen species in the form of highly damaging hydroxyl radicals and indirectly contributing to oxidative stress through free ferric ions via “Fenton reaction”. To counteract this oxidative stress, Salmonellae might have attempted to elevate the levels of other antioxidant enzymes leading to cellular adjustment towards metal stress environment^48–50^. Higher NPSH levels, GR, APOX and CAT enzyme activities were observed in CdA cells in comparison to CdunA cells (Table S1). However, SOD and GSH levels were found to be decreased in CdA Ty2 cells as compared to CdunA Ty2 cells (Table S1), thus co-relating with the zymographic analysis of SOD.

**Figure 5:**
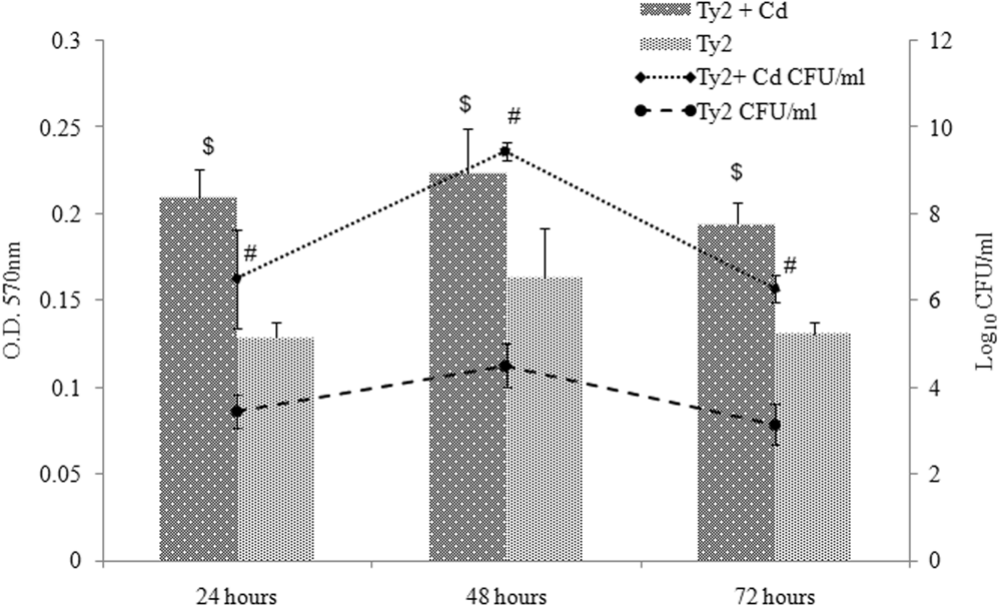
Quantification of biofilm formed in terms of optical density at 595 nm and log_10_ CFU/ml on polystyrene plates. The values were expresses as mean ± standard deviation. (^$^*p* < 0.01 versus normal cells and ^#^*p* < 0.05 versus normal cells).

To deepen our investigation on cadmium interference with other phenotypic characters of Salmonellae, biofilm formation potential and intracellular survival ability of CdA and CdunA cells were studied. After 24 and 48 h of biofilm formation, a significant increase (*p* < 0.01) in the OD at 600 nm was observed in CdA Ty2 cells in comparison to CdunA cells. Similarly in adapted cells during the three subsequent days, increase (*p* < 0.05) in log bacterial cells count was observed with respect to normal unadapted cells (Fig. 5). This observation is strengthened by many reports which suggest involvement of persistent bacterial population and metal sequestration in the extracellular matrix as an escape strategy of the biofilm cells^51^. All these observations prompted us to look for macrophage intracellular survival of CdA Salmonellae. The assay revealed increased survivability of CdA as compared to CdunA serovar Typhi Ty2 cells, as mean percentage of intracellular killing in CdA Salmonellae was found to be significantly decreased (*p* < 0.05) at 30, 60 and 90 minutes. On the other hand, comparatively increased killing was observed in CdunA serovar Typhi Ty2 cells (Table S2). Increased intracellular survivability by counteracting the oxidative stress mounted by macrophages may be correlated with the enhanced biofilm potential and increased antioxidant levels observed in the pathogen.

**Figure 6:**
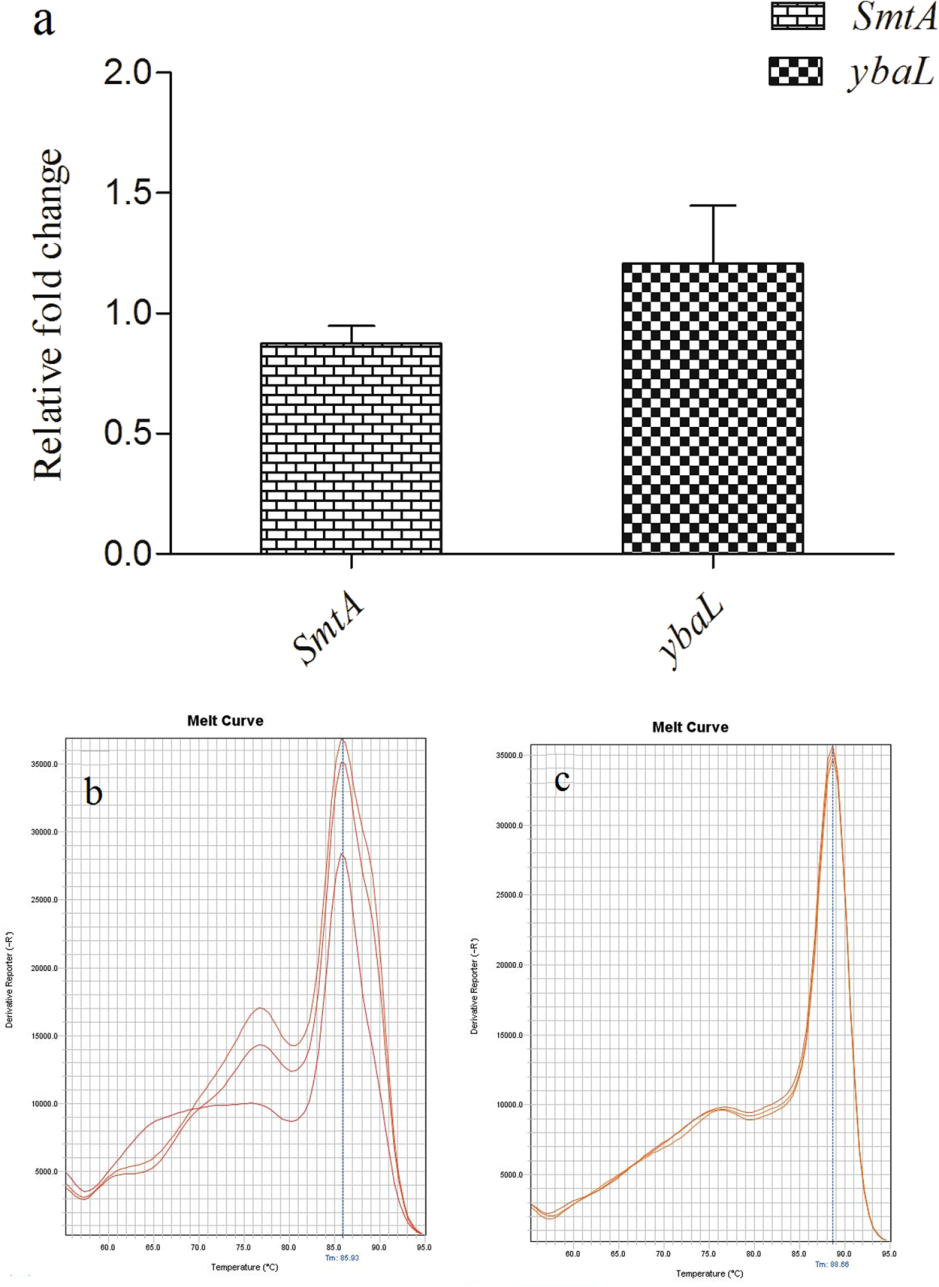
**(a)** Relative fold change in the gene expression of *smtA* and *ybaL* genes. The values are expressed as mean ± standard mean error. **(b**,**c)** Melt curves for *smtA* and *ybaL*.

*q*PCR analysis of CdA Ty2 strain revealed upregulated expression of *smtA* (encodes for bacterial metallothioneins, low molecular weight cysteine rich proteins, that can bind to cadmium and other heavy metal ions) with relative fold increase corresponding to 0.87 ± 0.07 in comparison to its expression in CdunA Ty2 cells (Fig. 6a) indicating bacterial adaptation to cadmium by sequestration^52^. The amplification of *smtA* gene observed in the present study confirmed its role in conferring resistance to heavy metal ions including cadmium, as reported earlier in *Salmonella enterica*^53,54^. In addition, the expression of putative metal transporter gene *ybaL*, was also found to be significantly altered in CdA Ty2 cells with relative fold increase corresponding to 1.21 ± 0.24 with respect to the CdunA Ty2 cells (Fig. 6a). *ybaL* encodes for K^+^–H^+^ efflux pump which forms part of large family of cation: proton antiporter-2 (CPA-2) in Gram-positive, Gram-negative bacteria and other higher organisms^55,56^. Within the bacterial cells, cation transport is important in maintenance of its physiological state and potassium transporters in particular have been shown to be actively involved in stress resistance and pathogenesis of *Salmonella*. Therefore, above observations indicate the role of *ybaL* in the pathogenesis of CdA cells, owing to its indirect link between type three secretion system of Salmonella Pathogenicity Island-1 (TTSS of SPI-1) and increased potassium efflux in Salmonellae as reported by Liu *et al*.^56^.

Considering all the observations of the present study, presence of cadmium inside the cells altered the susceptibility profile of *S*. *enterica* serovar Typhi towards the ampicillin, ciprofloxaxin, chloramphenicol and ceftizoxime, leading to an increase in MIC values suggesting that the sensitive isolates became resistant and resistant became more resistant as per their MICs. In addition to the increase in MIC values, accumulation of cadmium caused morphological, biochemical and physiological alterations that might be related to antimicrobial resistance of *S*. *enterica* serovar Typhi. The CdA Ty2 Salmonellae lowered down the expression of porin proteins, thereby restricting further entry of both metals and antibiotics. In response to these changes, the CdA Ty2 cells also responded by raising cellular antioxidants (other than SOD and GSH) and enhancing biofilm formation potential as a part of “survival strategy” thereby surviving the macrophage intracellular killing. The CdA Ty2 cells thus might defend themselves from metal insult by over-expressing the metal binding proteins and metal transporters in order to maintain the cellular physiology. This suggests involvement of co-regulatory response as a mechanism of metal induced antibiotic resistance in adapted cells^3^. Our future studies are being focussed on detailed proteomic exploration of CdA Salmonellae for identification of suitable protein inhibitor(s) in order to disarm the phenotypic expression involved in co-selection of metal and antibiotic resistance.

## Electronic supplementary material

Supplementary Information
