# Author Correction: Augmented antibiotic resistance associated with cadmium induced alterations in *Salmonella enterica* serovar Typhi

**DOI:** 10.1038/s41598-023-30981-6

**Published:** 2023-03-16

**Authors:** Ujjwal Jit Kaur, Simran Preet, Swaranjit Singh Cameotra, Praveen Rishi

**Affiliations:** 1 Department of Microbiology, Panjab University, Chandigarh, India; 2 Department of Biophysics, Panjab University, Chandigarh, India; 3 Institute of Microbial Technology, Chandigarh, 160036 India; 4 Present Address: Sas Polyclinic, Perth, Mohali, India

Correction to: *Scientific Reports* https://doi.org/10.1038/s41598-018-31143-9, published online 24 August 2018

This Article contains errors in the author list, where Swaranjit Singh Cameotra is inadvertently omitted.

As a result, the Author Contributions section should read:

P.R. conceived, organized and finalised the study. U.J.K. performed the experiments, collected the data. S.P. and U.J.K. carried out the macrophage intracellular survival assay. U.J.K and S.S.C carried out the FAAS related studies. U.J.K., P.R., S.P. interpreted the data and drafted the manuscript. All authors have critically reviewed and approved the final manuscript.

In addition, the Acknowledgements should read:

The authors acknowledge the help provided by the Instrumentation laboratory, IMTECH, Chandigarh for carrying out FAAS studies. The authors express their gratitude to Dr. Kanthi Kiran Kondepudi, Scientist-C, National Agri-Food Biotechnology Institute, Mohali, for providing assistance to carry out proteomics related work. The consumables were procured through funds provided by the ICMR project grant [No. 5/3/3/9/2013-ECD-I] provided to Professor Praveen Rishi.

The corrected Supplementary Information file is linked to this correction notice.

## Supplementary Information

Supplementary Information.

