# Supplementary material for: Author Correction: Augmented antibiotic resistance associated with cadmium induced alterations in Salmonella enterica serovar Typhi
Source: Sci Rep. 2023 Mar 16;13:4390. doi: 10.1038/s41598-023-30981-6 (PMC10020542; doi:10.1038/s41598-023-30981-6)
Supplement: Supplementary file 1 — Supplementary Information. [file 41598_2023_30981_MOESM1_ESM.docx]

**Augmented antibiotic resistance associated with cadmium induced alterations in *Salmonella enterica* serovar Typhi**

Ujjwal Jit Kaur^1^, Simran Preet^2^, Swaranjit Singh Cameotra^3^, Praveen Rishi^1^

^1^Department of Microbiology, Panjab University, Chandigarh

^2^Department of Biophysics, Panjab University, Chandigarh

^3^Institute of Microbial Technology, Chandigarh, 160036, India and presently Director, SAS Polyclinic, Perth, Mohali, India

**Fig. S1**- Graph representing effect of cadmium chloride supplementation on the growth of serovar Typhi Ty2 (in terms of O.D.600nm and CFU/ml). Values are expressed as mean ± standard deviation of three individual values.

After 24 hours, the MIC value of cadmium chloride was found to be 0.6mM, where the MIC of metal was considered to be the minimum concentration at which no growth occurred (Summers and Silver, (1972). Therefore, 0.5mM CdCl2 was taken as the sub-MIC value at 24 hours.

The growth estimation in order to assess adaptability of serovar Typhi Ty2 to cadmium choride, was recorded for 72 hours. After a period of 72 hours, 2 log units (O.D. 600nm= 0.128) of serovar Typhi cells survived in nutrient broth supplemented with 0.5mM CdCl_2_. Therefore, this sub-MIC value was further used for adapting the serovar Typhi to cadmium chloride.

**Figure. S2-** Graph showing collectively MIC change (in terms of %age) in all the strains of *Salmonella enteric*a serovar Typhi

**Figure. S3-** SDS-PAGE analysis of different protein fractions from CdunA serovar Typhi Ty2 cells and CdA Ty2 cells. Lane M- Broad range protein molecular weight marker; Lane 1,3,5- CYT, IM, OM fractions from CdunA serovar Typhi Ty2 cells and Lane 2,4,6- CYT, IM, OM fractions from CdA serovar Typhi Ty2 cells. Arrows indicate the changes observed.

**
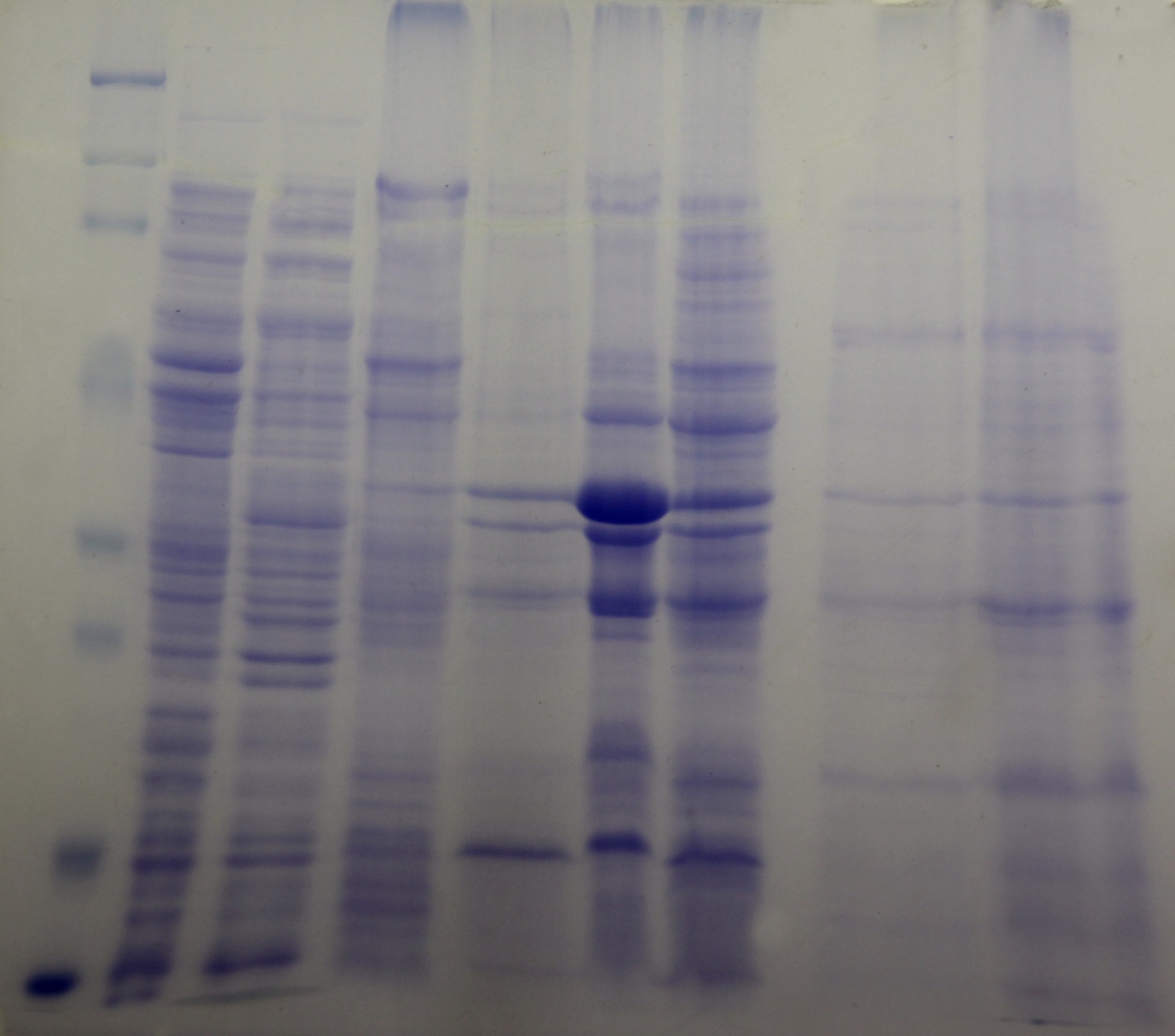
**

M 1 2 3 4 5 6

**Figure. S4-** SDS-PAGE analysis of different protein fractions from clinical serovar Typhi 25C cells and cadmium adapted 25C cells. Lane M- Broad range protein molecular weight marker; Lane 1,3,5- CYT, IM, OM fractions from normal serovar Typhi 25C cells and Lane 2,4,6- CYT, IM, OM fractions from cadmium adapted 25C cells. Arrows indicate the changes observed. (Complete figure, without any contrasting or modification is given on the next page).


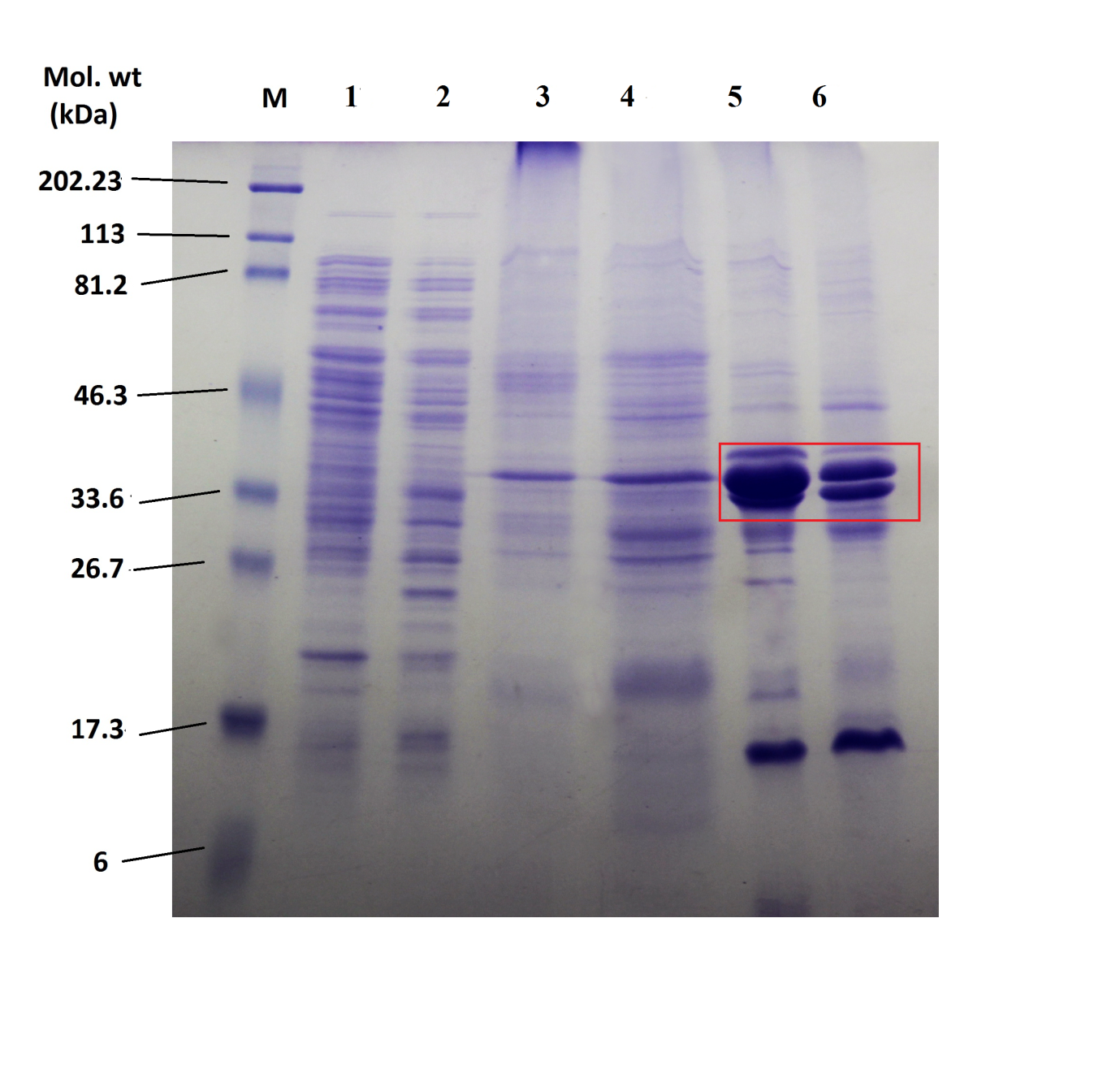


**
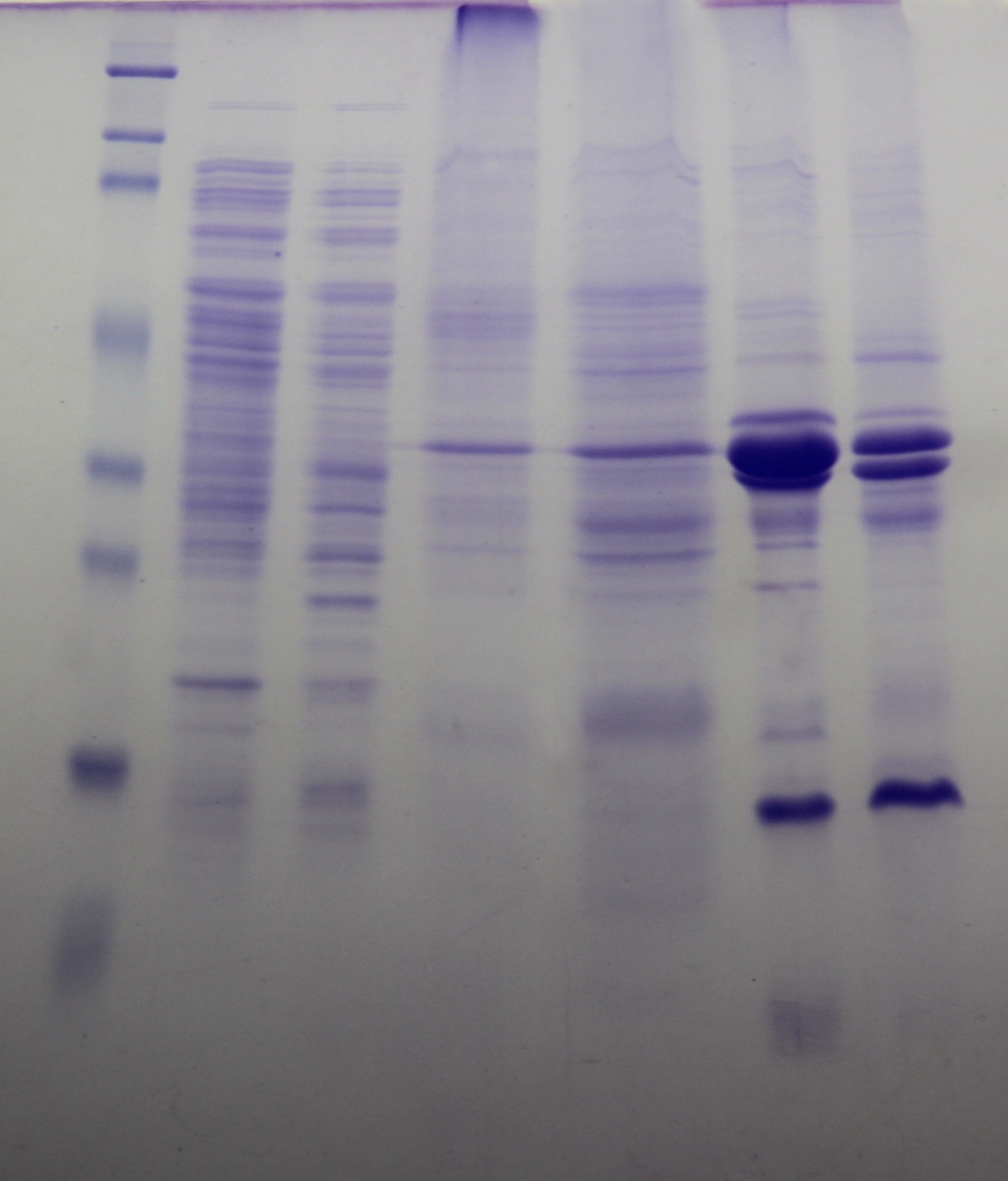
**

Figure. S4 - original figure

M 1 2 3 4 5 6

**Fig. S5** - SDS-PAGE analysis of different protein fractions from clinical serovar Typhi 12G cells and cadmium adapted 12G cells. Lane M- Broad range protein molecular weight marker; Lane 1,3,5- CYT, IM, OM fractions from normal serovar Typhi 12G cells and Lane 2,4,6- CYT, IM, OM fractions from cadmium adapted 12G cells. Arrows indicate the changes observed. . (Complete figure, without any contrasting or modification is given on the next page).


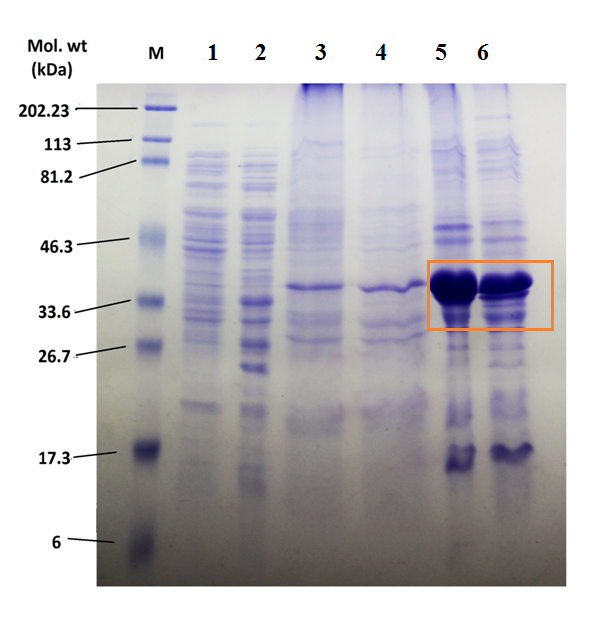


Figue. S5- original figure


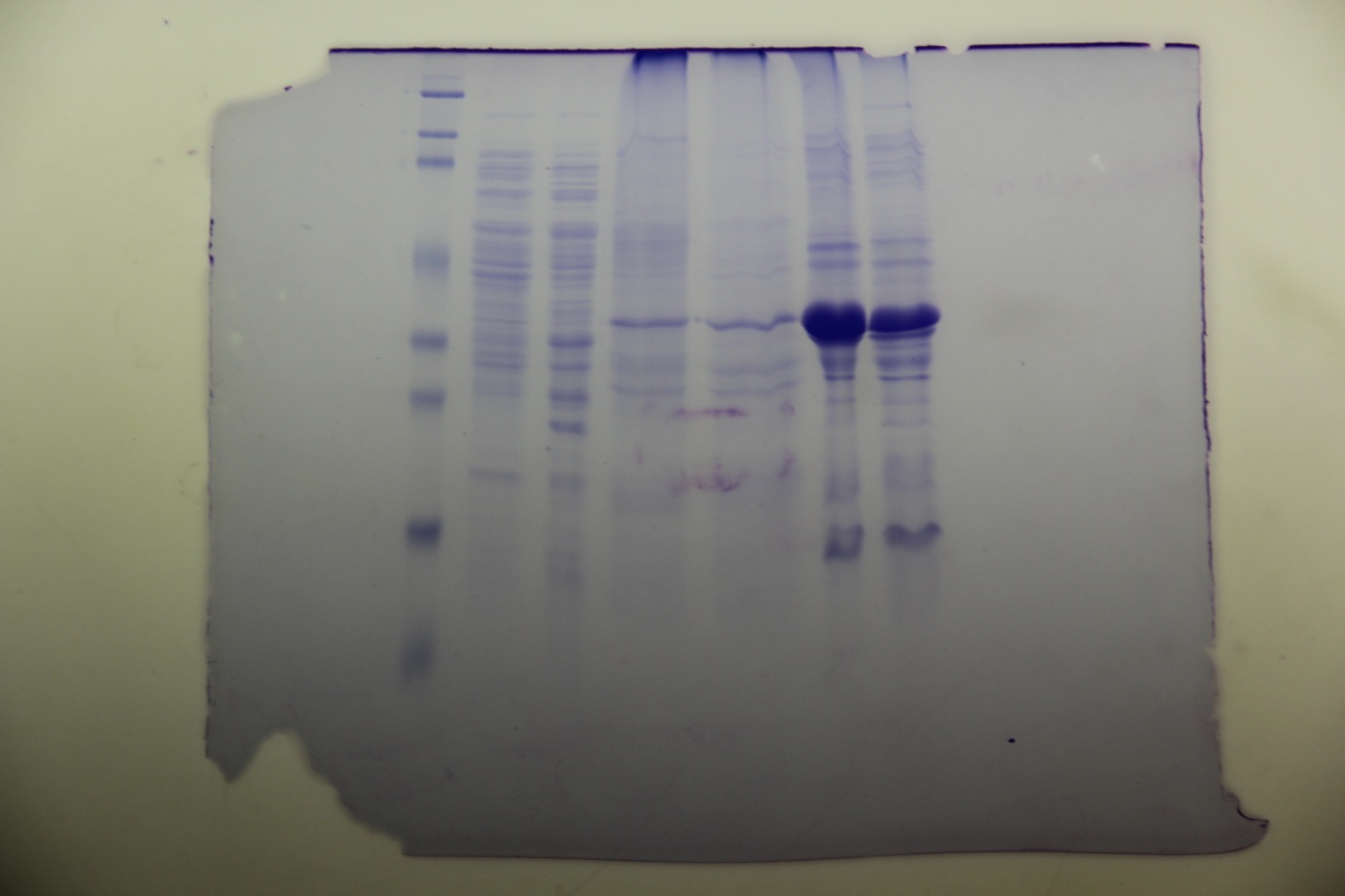


M 1 2 3 4 5 6

**Figure. S6**- Cadmium dependent variation of SOD enzymes. Serovar Typhi 25C and 12G grown with and without cadmium supplementation, whole cell lysates (WCL) were loaded on native PAGE gel and stained to visualise different isoforms of SOD. Lane 1,3 - normal serovar Typhi cells and Lane 2,4 - cadmium adapted serovar Typhi cells.

**
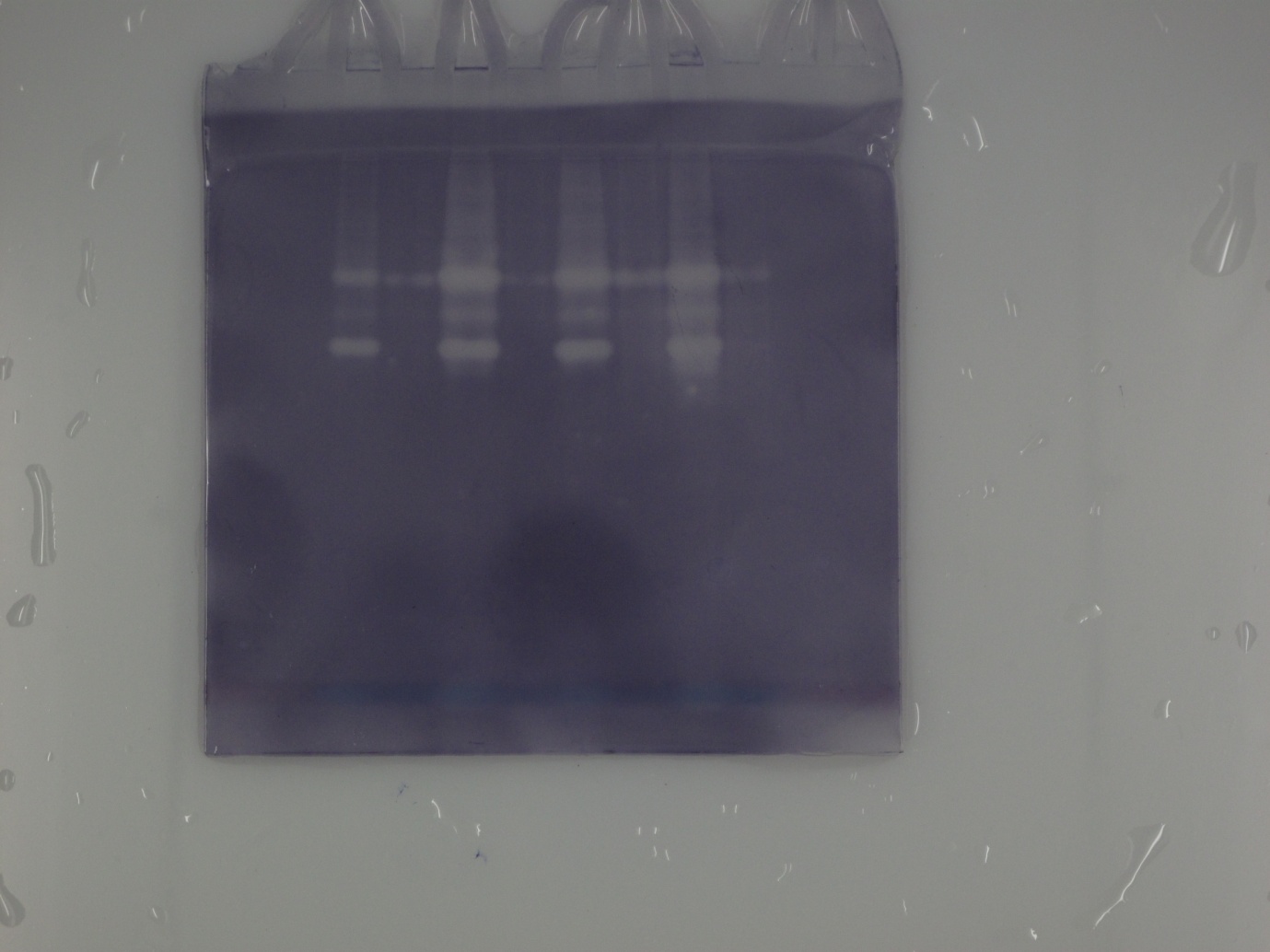
**

25C

12G

1 2 3 4

Table.S1- Levels of different antioxidants in cadmium adapted as well as unadapted *Salmonella enterica* serovar Typhi Ty2 cells

| Strains | **SOD**  **(U mg^-1^ FW)** | **Reduced**  **GSH** | **Ascorbate peroxidase**  **(U g^-1^ FW)** | **Catalase**  **(U g^-1^ FW)** | **NPSH**  **(µmol g^-1^ FW)** | **Glutathione reductase**  **(U g^-1^ FW)** |
| --- | --- | --- | --- | --- | --- | --- |
| *S*. Typhi Ty2 | 0.40 ± 0.13 | 10.15± 0.72 | 0.55 ± 0.03 | 0.10 ± 0.03 | 16.18 ± 1.08 | 0.484 ± 0.01 |
| Cadmium adapted  *S*. Typhi Ty2 | 0.13 ± 0.02 | 9.27 ± 0.354 | 0.76 ± 0.02 | 0.39 ± 0.03 | 86.43 ± 0.37 | 0.86 ± 0.04 |

| **Duration of intracellular incubation** | **Cfu (mean ± SD) (*N_t_*)** | | **Mean percentage killing**  **[(*N_0_ - N_t_/ N_0_*) x 100** | |
| --- | --- | --- | --- | --- |
|  | **CdunA Salmonellae** | **CdA Salmonellae** | **CdunA Salmonellae** | **CdA Salmonellae** |
| 0 (*N_0_*) | 6x10^9^ | ^-^ |  |  |
| 30 | 52x10^8^±0.71 | 58x10^8^±1.10 | 12.7%±1.18% | 3.78%±1.83* |
| 60 | 46x10^8^±1.36 | 55x10^8^±0.95 | 22.96%±2.28% | 8.36%±1.60%* |
| 90 | 22x10^8^±1.50 | 30x10^8^±0.91 | 64.14%±2.50% | 49.32%±1.67%* |

Table.S2- Intracellular killing of cadmium adapted (CdA) and cadmium unadapted (CduA) *Salmonella* Typhi Ty2 cells by mouse peritoneal macrophages

*p*<0.05 intracellular killing in CdA cells as compared to CdunA cells

References-

1. Summers, Α.Ο., & Silver, S. Mercury resistance in a plasmid-bearing strain of *Escherichia coli*. *J Bacteriol* **112**, 1228-1236 (1972).
